# The Effect of Primary and Middle School Teachers’ Problematic Internet Use and Fear of COVID-19 on Psychological Need Thwarting of Online Teaching and Psychological Distress

**DOI:** 10.3390/healthcare9091199

**Published:** 2021-09-11

**Authors:** Jian Yi, I-Hua Chen, Chung-Ying Lin, Cheng-Chieh Li, Xiao-Ling Liao, Zhi-Hui Wei, Jeffrey Hugh Gamble

**Affiliations:** 1 College of Education, Capital Normal University, Beijing 100048, China; yijian5436@126.com; 2 Chinese Academy of Education Big Data, Qufu Normal University, Qufu 273165, China; 3 Institute of Allied Health Sciences, National Cheng Kung University Hospital, College of Medicine, National Cheng Kung University, Tainan 701, Taiwan; cylin36933@gs.ncku.edu.tw; 4 Department of Public Health, National Cheng Kung University Hospital, College of Medicine, National Cheng Kung University, Tainan 701, Taiwan; 5 Department of Occupational Therapy, College of Medicine, National Cheng Kung University, Tainan 701, Taiwan; 6 School of Education, Zhaoqing University, Zhaoqing 526061, China; 2019013013@zqu.edu.cn; 7 International College, Krirk University, Bangkok 10220, Thailand; ndxlfzr@126.com; 8 Development and Research Department, Shanghai Open University, Shanghai 200433, China; huixuanwei@163.com; 9 Department of Foreign Languages, National Chiayi University, 85 Wenlong Village, Minhsiung County, Chiayi 62103, Taiwan

**Keywords:** problematic internet use, psychological need thwarting, psychological distress, online teaching, COVID-19, online survey, structural equation modeling, primary and middle school teachers

## Abstract

Problematic Internet use (PIU) is a risk factor for psychological distress during COVID-19, as teachers are a psychologically vulnerable population. We explored the role of PIU in terms of primary and middle school teachers’ fear of COVID-19 and psychological need thwarting (PNT) of online teaching. We empirically evaluated the relationships among these research variables in explaining teachers’ psychological distress during COVID-19. Online survey data were collected from 9030 teachers. A high proportion of participants demonstrated psychological distress: depression (20.4%), anxiety (26.4%), and stress (10.2%). Structural equation modeling was used to test our proposed conceptual model, wherein PIU behaviors served as predictors, mediated by fear of COVID-19 and PNT of online teaching, for teachers’ psychological distress. With ideal model fit, the results of the path coefficients indicated that PIU behaviors were associated with fear of COVID-19 (*p* < 0.001); fear of COVID-19 and PNT of online teaching were associated with psychological distress (*p* < 0.001); and fear of COVID-19 was also positively associated with PNT of online teaching (*p* < 0.001). PSU and PSMU had an indirect positive effect on psychological distress through the mediator of fear of COVID-19 and PNT of online teaching. As such, we suggest that school administrators pay greater attention to teachers’ psychological needs through efforts to enhance teachers’ autonomy and relatedness from interpersonal relationships, alleviating PNT of online teaching. Our PNT of online teaching scale may also serve as a contribution for further research and practice.

## 1. Introduction

The pandemic caused by the novel coronavirus disease 2019 (COVID-19) has resulted in quarantines and isolation at home in many countries. In addition to the physical effects of the pandemic, scholars are paying increasing attention to the effects on individuals’ mental health, including stress caused by restrictions on movement, COVID-19 anxieties, and fear of infection [1,2,3,4]. Scholars and medical professionals often refer to these potentially long-lasting psychological impacts as a “second wave” of the pandemic [5].

### 1.1. Primary and Middle School Teachers as a Vulnerable Population

In China, primary school includes first–sixth grades (with children from 6 to 11 years old), while middle schools include seventh–ninth grades (with children from 12 to 14 years old). The impacts of COVID-19 on specific populations whose work requires substantial changes have not been sufficiently evaluated. Specifically, given the closure of school campuses and the implementation of online teaching [6,7,8], teachers are a vulnerable population in terms of mental health risk factors. Preliminary analysis of demographic factors related to the prevalence of anxiety among teachers in China during COVID-19 demonstrated that primary school teachers are at greater risk as compared to high school teachers [9]. Several factors make teachers vulnerable to the impact of COVID-19 and online teaching, including strains on teacher–learner relationships during interrupted learning, uncertainty regarding current and future responsibilities, and technical and logistic challenges resulting from the transition from classroom to home-based teaching [10].

Researchers have evaluated the impact of COVID-19 on psychological well-being in educational contexts but have tended to focus on universities, reporting negative biopsychosocial effects at the tertiary (higher education) level [11] or mental health issues for children and adolescents following school closures [12]. While these studies highlight the negative impacts of COVID-19, further research focusing on primary and middle school teachers as a vulnerable population is lacking.

Primary and secondary school students spend significantly more time learning online as compared to university students [13]. As a result, the amount of online teaching is associated with the prevalence of emotional problems among teachers [10]. Online teaching negatively influences teachers’ mental health since most teachers were not well-prepared with technological skills required for online teaching [6,7,8,10], with school closures and online teaching requirements announced without sufficient time to prepare. Online teaching requires information and communication technology (ICT) literacy and experience in online teaching design [14] in order to successfully teach online. In a survey of online teaching by Chinese primary and secondary school teachers, less than half of the respondents reported sufficient ICT skills to share digital teaching resources, conduct collaborative teaching, or use learning management systems to evaluate student learning outcomes [6]. As a result, 31.3% of teachers were not willing to continue online teaching after the epidemic [6]. Similar results were found among Indonesian primary school teachers, where 80% of teachers were dissatisfied with the efficiency of online teaching [8]. Given this dissatisfaction, it is critical to evaluate the role of online teaching in terms of teachers’ psychological well-being.

### 1.2. Online Teaching and Teacher Psychological Well-Being

In addition to the studies on teachers’ dissatisfaction and negative emotion in response to online teaching demands mentioned above, research has discussed how online teaching activities result in additional work-related burdens, pressure, and excessive time expenditures [10,15,16]. However, there is a lack of large-scale empirical studies evaluating impacts on the psychological well-being of teachers. Existing research tends to emphasize demographic factors, such as sex or school type, without considering the influence of online teaching [9]. As such, risk factors related to teachers’ psychological distress during COVID-19 in the context of online teaching are in need of empirical evaluation. 

One potential issue related to online teaching is the role of problematic Internet use (PIU). In evaluating risk and protective factors related to the online teaching experience of Italian high school teachers during COVID-19, Truzoli et al. [17] noted some excessive time spent online, consistent with PIU in the general population. PIU is characterized by addictive behaviors, such as preoccupation (salience), changes in mood, development of tolerance (and the need for increased amounts of online activity), withdrawal effects, interpersonal conflicts resulting from online use, and relapse following a period of abstinence [18]. PIU frequently involves addictive use of smartphones (PSU) and social media (PSMU), which are the two key measures of PIU adopted by the current study and which have demonstrated negative effects on health outcomes [19]. While recent findings [17] found some evidence of PIU among high school teachers, the current study evaluates primary and middle school teachers as a vulnerable population in terms of relatively more intensive demands for designing, conducting, and evaluating learning online, resulting in higher levels of required Internet use (or mobile device use) over an extended period of time [6,8,10]. Since most primary and middle school teachers also serve as home-room teachers [20], mobile devices, such as smartphones, are frequently required to communicate with parents. However, given the Internet access provided by smartphones, PSU negatively impacts mental health, including increased Internet addiction [19,21] and attention-related disorders [22].

Moreover, teachers in environments with immediate Internet access may also receive more frequent information on COVID-19, as many social media applications automatically provide breaking news (such as pandemic-related announcements). For example, Weibo, the dominant microblogging website in China with more than 500 million users, automatically lists major social news events after logging in, forcing users to view headlines related to breaking news stories, particularly concerning COVID-19. Moreover, teachers are also more likely to take the initiative to search for information on COVID-19 using social media [2,23].

As a result of teachers’ exposure to online content, fear of COVID-19 may develop and worsen. Fear of COVID-19 is a recent development that is strongly related to PIU and the concept of “cyberchondria” or the repeated and addictive use of online health information during COVID-19, resulting in increased anxiety and distress regarding one’s health [24]. In fact, PIU related to COVID-19 information results in increased feelings of fear or threat regarding the virus and difficulties in handling overwhelming news [25]. Unfortunately, whether teachers are passively (via automatic updates) or actively (through intentional searching) exposed to COVID-19 related social media, negative emotions may be induced for two main reasons: (a) disturbing information may deepen one’s anxiety [4,23,26]; and (b) when updated information is not immediately available, individuals will tend to search for information, forming a negative emotional feedback loop [3]. This type of negative emotion related to COVID-19 can be characterized as fearfulness, with fear of COVID-19 clearly related to mental health issues, including anxiety and depression [27].

In the context of online teaching, potentially increased PIU behaviors and fear of COVID-19 serve as risk factors for psychological distress. Psychological distress, in this study, is defined as feelings of stress, anxiety, and depression [28]. The impact of fear of COVID-19 on anxiety and depression has been supported by a recent cohort study of adults, finding an increase of between 6.6- and 7.4-fold in depression and anxiety [29]. Existing empirical findings suggest that PSU and PSMU serve as two critical factors related to teachers’ fear of COVID-19 [30] and overall psychological distress during COVID-19 [3,23,31,32,33,34]. Thus, the role of PSU and PSMU in terms of fear of COVID-19 and psychological distress requires further empirical evaluation and modeling to understand the relationships among these important variables. Moreover, the emerging perspective of psychological need thwarting has the potential to assist in further evaluating how online teaching itself contributes to teachers’ psychological well-being.

### 1.3. Psychological Need Thwarting of Online Teaching

Psychological need thwarting of teachers is an emerging construct in health research which adds to an improved understanding of how the frustration, as opposed to satisfaction, of teachers’ needs for autonomy (such as inhibited decision-making), competence (including situational incompetence), and relatedness (including rejection) impacts teachers’ and students’ mental health and performance [35]. Based on previous studies, it is evident that online teaching is viewed negatively by teachers, with very low satisfaction and intention to engage in online teaching [6,8], suggesting that thwarting of teachers’ psychological needs occurs during COVID-19. Although most teachers consider online teaching to be necessary, given the preventative effect of distance learning on the spread of the virus [10], teachers’ motivation has suffered due to a lack of direct interaction with students [7]. Unfamiliarity with online teaching and low teaching motivation may be reflected in teachers’ lack of autonomy and competence in online teaching. Moreover, during the epidemic, teachers are isolated at home, restricting their need for relatedness [10]. According to self-determination theory (SDT), autonomy, competence, and relatedness are three basic psychological needs impacting motivation [36,37]. When these three psychological basic needs are not satisfied, frustration occurs, resulting in a phenomenon called psychological need thwarting (PNT) [38,39]. This study proposes a novel construct based on the PNT within the context of online teaching: PNT of online teaching.

We believe that the PNT of online teaching can provide a new perspective for interpreting the mechanisms contributing to mental health issues among teachers during the epidemic. We suggest that PNT of online teaching may contribute to teacher’s psychological distress since higher PNT is associated with professional burnout [35,40], a construct closely related to psychological distress [41]. However, the effects of PNT of online teaching require further examination, including the relationships among PNT of online teaching, mental health, and PIU. As such, modeling of these key factors can provide more clear representations of the relationships among the variables of PIU, fear of COVID-19, PNT of online teaching, and psychological distress. The following sub-section introduces our conceptual model, which has the potential to explain the relationships among the variables evaluated in this study.

### 1.4. Conceptual Model

Recent research provides a perspective on the relationships between PIU and psychological distress. Namely, problematic or addictive Internet use behaviors can be considered as risk factors predictive of psychological distress [19,42,43]. For example, in Chen et al.’s longitudinal study using latent growth modeling, evidence more strongly supported a causal interpretation of PIU as a predictor of psychological distress among Hong Kong university students, with PSU and PSMU causing psychological distress [19]. It is probable that the association between problematic Internet use behaviors and psychological distress may be even stronger during the COVID-19 pandemic, given the results of Chen et al.’s study comparing the magnitude of the association of Internet use behaviors with psychological distress between measures taken pre-COVID-19 and during the COVID-19 outbreak [44], which found that PSU and PSMU were only positively associated with psychological distress during the COVID-19 outbreak but not prior to the outbreak. One explanation is that individuals restricted to their homes may rely on social media to receive updated COVID-19 information, with social media potentially providing false or exaggerated reports [4]. In addition, negative emotions expressed online negatively influence psychological well-being through “(mis)information overload” [4]. 

**Figure 1:**
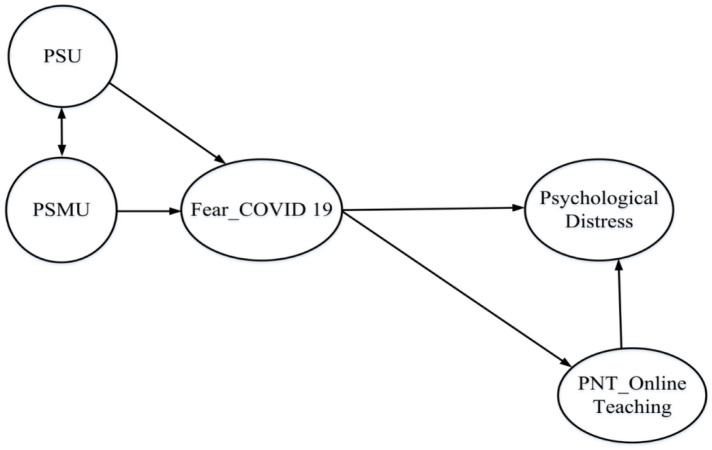
Conceptual Model.

In our conceptual model (Figure 1), all hypothesized relationships are positive. This model is based on previous studies suggesting PIU leads to psychological distress [4,19,42,43,44,45]. As such, PSU and PSMU serve as explanatory variables significantly influencing teachers’ psychological distress (the dependent variable). Recent research suggests that, during COVID-19, fear of COVID-19 mediates PIU and psychological distress [24]. Since teachers use smartphones and social media more frequently during the outbreak, partially due to online teaching, increased exposure to information can result in greater fear of COVID, resulting in general psychological distress, a mechanism supported by research on the impact of PSMU on psychological distress [2]. Studies of emotion suggest that fear influences information integration and working memory, limiting one’s conscious decision making and preventing adaptive behaviors [46]. As such, increase fear of COVID-19 may restrict teachers’ autonomy in online teaching, thereby directly contributing to PNT of online teaching. Since PNT is positively associated with teacher burnout (including anxiety and depression) [35,40], the model hypothesizes that PNT of online teaching will be positively related to mental health problems since PNT has been demonstrated to serve as a proxy for psychological distress [41,47].

### 1.5. Aims of the Present Study

Although existing COVID-19 research emphasizes the association between PIU and psychological distress [2,3,23,33,44], there is a clear gap in the literature in terms of the following: (a) studies on the vulnerable population of primary and middle school teachers whose work and lifestyle are disproportionately affected by COVID-19 restrictions and (b) the novel construct of PNT of online teaching, based on emerging research in the area of teacher psychological well-being [35]. As such, in the present study, we conducted a cross-sectional survey investigating the prevalence of psychological distress among primary and middle school teachers in mainland China. More importantly, in order to further understand the relationships among problematic Internet use behaviors (i.e., PSU and PSMU), PNT of online teaching, fear of COVID-19, and psychological distress, we developed and examined a conceptual model which offers a plausible interpretation and implications, supported by empirical findings. As such, this study contributes to both theory and practice by evaluating the associations among PIU, PNT of online teaching, fear of COVID-19, and psychological distress experienced by primary and middle school teachers. Implications for teaching and practice, based on a systematic and comprehensive evaluation of the conceptual model, are provided through the empirical evaluation of data from a large sample of teachers. In order to provide insights into the factors impacting teachers’ mental health during COVID-19, the following specific research questions are be addressed:

- What is the prevalence of psychological problems among the teachers sampled, including psychological distress, PNT of online teaching, fear of COVID-19, PSU and PSMU? (RQ1)
- Which relationships among variables are statistically significant? (RQ2)

## 2. Materials and Methods

### 2.1. Participants and Procedure

This study conducted a cross-sectional online survey of primary and middle school teachers between 25 May and 30 June 2020. Ethics approval for the study was provided by the Institutional Review Board (IRB) of the Jianxi Psychological Consultant Association (IRB ref: JXSXL-2020-J013). In order to collect data reflecting the period during which online teaching was adopted by school teachers, it was necessary to conduct the survey in a timely manner (before the end of the semester). This made it difficult to collect a comprehensive sample representing all provinces of mainland China. As such, a non-probability sampling strategy was adopted using an online survey, Sojump, a platform for online questionnaire collection. We selected one province each from western (Sichuan), central (Jiangxi), and eastern (Shandong) regions of mainland China, which contain half of the country’s population. The sample was deemed to be reasonably representative of the national population of primary and secondary school teachers when compared to available demographics (see Table 1). We first sought help from principals of primary and middle schools in Jiangxi, Sichuan, and Shandong, China. Subsequently, the principals who accepted our invitation provided the survey’s hyperlink to their school’s teachers. The online survey was voluntary and anonymous. Informed consent was obtained at the beginning of the survey. In total, 11,014 primary and middle school teachers completed the online survey.

As the Sojump platform required participants to complete all items, there were no missing data. However, not all participant data were utilized for subsequent analysis, as we only selected participants who had experience in online teaching during the COVID-19 outbreak. Filtering of responses was achieved through the use of one survey item, which asked, “During the epidemic, did you conduct online teaching (including online teaching, assigning and grading homework in the Internet environment, etc.)?” The screening was based on responses to this item, and only teachers with online teaching experience were included, with an effective sample of 9030 participants, including 5838 responses from primary school teachers (65%) and 3192 responses from junior high school teachers (35%), with an average age of 33.94 years. The majority of teachers were female (6563, 72.7%), from public schools (8671, 96%), served as home-room teachers (5225, 57.9%), and were from urban areas (5549, 61%). Further demographic data can be found in Table 1. Since there were no significant differences in the observed scores between primary and junior high school teachers on the target variables, we integrated the data from the two samples (primary and middle school teachers) during this stage of model evaluation.

### 2.2. Measures

Five validated psychometric instruments were used to evaluate teachers’ psychological distress, fear of COVID-19, problematic smartphone use, problematic social media use, and psychological need thwarting of online teaching. These instruments were administered as online questionnaires wherein participants were asked to self-evaluate themselves in terms of these constructs during a specific timeframe in which the COVID-19 outbreak was considered to be the most serious in terms of its impact on teachers and online teaching; namely, the school semester beginning in February 2020 and ending in June 2020. Apart from the scale for PNT of online teaching, which was developed and validated specifically for this study, all other scales have been well-developed and validated in the literature and widely adopted for research in the Chinese context.

#### 2.2.1. Psychological Distress

We used scores from the Depression, Anxiety and Stress Scale-21 (DASS-21) as indicators for psychological distress. The DASS-21 contains 21 items evenly divided into three subscales: depression, anxiety, and stress (each subscale consisting of seven items) [28]. All items were rated using a four-point Likert scale (from 0 to 3), with higher scores indicating higher levels of depression, anxiety, or stress. We summed all item scores for each subscale and multiplied the summed scores by two to facilitate a comparison with the scale’s cut-off values. According to the DASS-21 manual, multiplied scores higher than 10 (depression), 8 (anxiety), and 15 (stress) indicate a mental health problem [28]. Prior studies have demonstrated the sound psychometric properties of the Chinese version of DASS-21 [50,51] among mainland Chinese respondents. In the present study, the results of confirmatory factor analysis (CFA) revealed an ideal fit for the three-factor structure (χ^2^ = 4224.93.83 (*p* < 0.001), CFI = 0.996, NNFI = 0.996, RMSEA = 0.049, and SRMR = 0.028).The internal consistency of the Chinese DASS-21 instrument utilized in our study was high (ranging from 0.91 to 0.92 for depression, anxiety, and stress). Sample items for each subscale include: “I couldn’t seem to experience any positive feelings at all” (depression); “I was aware of dryness of my mouth” (anxiety); and “I tended to over-react to situations” (stress).

#### 2.2.2. Fear of COVID-19

The Fear of COVID-19 Scale (FCV-19S) was used to measure fear of COVID-19. The FCV-19S contains seven items scored on a five-point Likert-type scale (from 1 to 5). Higher scores on the FCV-19S indicate greater fear of COVID-19. FCV-19S was developed by Ahorsu et al. [52] and has sound psychometric properties. In the present study, the results of CFA were deemed to be acceptable (χ^2^ = 809.21 (*p* < 0.001), CFI = 0.990, NNFI = 0.983, RMSEA = 0.086, and SRMR = 0.042).The internal consistency of the FCV-19S in this study was 0.89, indicating good reliability. Sample items include: “I am most afraid of COVID-19” and “I am afraid of losing my life due to COVID-19”.

#### 2.2.3. Problematic Smartphone Use

The Smartphone Application-Based Addiction Scale (SABAS) was used to measure respondents’ level of PSU. Csibi et al. developed SABAS [53], which includes six items. SABAS measures a single construct: risk of addiction to smartphone applications. SABAS was used to assess general problematic smartphone/Internet use. SABAS items were scored on a six-point Likert-type scale (from 1 to 6), with higher scores indicating more problematic smartphone use. The psychometric properties of the Chinese version of SABAS have been established [54,55]. In the present study, the results of CFA were acceptable (χ^2^ = 578.83 (*p* < 0.001), CFI = 0.989, NNFI = 0.979, RMSEA = 0.088, and SRMR = 0.032). Internal consistency in the present study was 0.87, which was deemed acceptable. Sample items include: “My smartphone is the most important thing in my life” and “I tend to increasingly use my smartphone”.

#### 2.2.4. Problematic Social Media Use

The Bergen Social Media Addiction Scale (BSMAS) was adopted to measure PSMU. BSMAS was developed by Andreassen et al. [56] and includes six items scored on a five-point Likert-type scale (from 1 to 5), with higher scores indicating a greater risk of social media addiction. The Chinese version of BSMAS has sound psychometric properties [54,55]. In the present study, the results of CFA were acceptable, in that all indices met standard criteria (χ^2^ = 344.68 (*p* < 0.001), CFI = 0.994, NNFI = 0.988, RMSEA = 0.068, and SRMR = 0.032).The internal consistency of BSMAS in the present study was satisfactory (α = 0.88). Sample items include: “I spend a lot of time thinking about or planning on using social media” and “I use social media to forget about personal problems”.

#### 2.2.5. Psychological Need Thwarting of Online Teaching

According to our review of the literature, there is no published instrument available to measure the psychological need thwarting of online teaching. Therefore, we made slight modifications to the original content of the Chinese Psychological Need Thwarting of Teachers Scale (CPNTTS) [35] in order to reflect online teaching tasks. The CPNTTS includes three subscales: autonomy, competence, and relatedness [35]. The original CPNTTS includes nine items scored on a seven-point Likert-type scale (from 1 to 7). Based on the original scale, revisions were made to adapt the content to assess the PNT of online teaching. The revisions were conducted by two of the original authors of the CPNTTS scale, who are professors in the field of educational psychology and who have published works in teacher psychology, SDT, and the psychological impacts of COVID-19. Expert validity was established through further review by a third professor with a similar academic background. Face validity was reached through the collaboration of two primary and middle school teachers who had experience with online teaching. A comparison of items from the original CPNTTS and the revised PNT of online teaching scale is provided in the Supplementary Materials in Table S1.

Although the original CPNTTS has demonstrated sound psychometric properties [35], we conducted a strict re-examination of the reliability and validity of the new “PNT of Online Teaching Scale” since modifications were made for the purposes of the present study. First, we randomly selected half of the data from the total sample of 9030 in order to conduct exploratory factor analysis (EFA). The Kaiser-Meyer-Olkin statistic (0.867) and Bartlett’s test of sphericity (*p* < 0.001) indicated the selected data was suitable for conducting EFA. Table S1 in the Supplementary Materials shows the results of EFA for the Psychological Need Thwarting of Online Teaching Scale. First, three factors were extracted (as confirmed by the results of a scree plot, shown in Figure S1), with the eigenvalues of each factor higher than 1. Second, the total explained variance was 60.50%. Third, the factor loading of each item corresponded to the original CPNTTS item, with each value higher than 0.50. Since these EFA results were very satisfactory, we directly used data from the whole sample to conduct CFA. The results confirmed that the factor structure of the revised version was consistent with the original CPNTTS scale (i.e., a three-factor structure), with the results of CFA demonstrating satisfactory fit on relevant indices (CFI = 0.966, NNFI = 0.955, RMSEA = 0.09, and SRMR = 0.05). Moreover, factor loadings were between 0.63 and 0.88, which was deemed acceptable. The Cronbach’s α for the sub-scales were as follows: autonomy (0.78), competence (0.84), and relatedness (0.88). Based on the above results, the revised version of CPNTTS was deemed to have acceptable validity and reliability.

#### 2.2.6. Demographic Information

In addition to age, educational system (primary or middle school), sex, and years of work experience, two key demographic variables were also evaluated: status as a home-room teacher and previous online teaching experience before the school hiatus (when schools closed and online courses commenced).

### 2.3. Statistical Analyses

We first provided descriptive statistics on our target variables (i.e., psychological distress, fear of COVID-19, PSU, PSMU, and PNT of online teaching). We also further reported the proportion of respondents who reported PSU, PSMU, and psychological distress, including depression, anxiety, and stress, based on the cut-off values of SABAS [57], BSMAS [58], and DASS [28]. Furthermore, the results of Pearson correlations were also provided. 

In order to provide systematic modeling of the data, structural equation modeling (SEM) was used to simultaneously evaluate the relationships/associations among variables, including an analysis of measurement error, inclusion of control variables, and determination of model fit. Specifically, we used SEM with LISREL 8.80 to first test the measurement model (focusing on convergent and discriminant validity) and then evaluate the full structural equation model. Diagonally weighted least squares (DWLS) was used to estimate parameters since DWLS is appropriate for estimating non-normally distributed data [59]. The mean scores for subscales were used as indicators for psychological distress (DASS-21) and PNT of online teaching, given that the validity of the three-factor structure of both two instruments was supported (see Section 2.2.1 and Section 2.2.5). We followed the approach of homogeneous parceling wherein each parcel is comprised of items (using mean scores) that load onto the same first-order factor [60]. Namely, in order to address the hierarchical nature, mean scores for sub-scales from the DASS-21 and PNT of online teaching instrument were used as indicators for the latent variables of psychological distress and PNT of online teaching, while the original items were used as indicators for other latent variables with one-factor structure (i.e., fear of COVID-19, PSU, and PSMU). Model fit criteria included χ^2^, comparative fit index (CFI), non-normed fit index (NNFI), root mean square error of approximation (RMSEA), and standardized root mean square residual (SRMR). RMSEA values of 0.06 or lower, SRMR values of 0.08 or lower, CFI values of 0.95 or higher, and NNFI values of 0.95 or higher were considered acceptable [61]. Regarding convergent and discriminant validity, in addition to fit indices, other important psychometric indices were adopted [62,63]: factor loading, average variance extracted (AVE), and construct reliability (CR). The suggested cut-off points for convergent validity were: (i) factor loading of each indicator ≥0.50; (ii) latent variables of each AVE ≥0.50; (iii) CR ≥0.70. To support discriminant validity, the square root of each AVE should exceed the correlations among latent variables for each pair. Finally, since the data were collected from the same source, common method bias was evaluated through Harman’s one-factor test [64].

## 3. Results

### 3.1. Participant Characteristics

Table 1 includes key participant characteristics. The mean age of respondents was 33.94 years (SD = 8.81), with 6563 female teachers (72.7%). Most participants had over 10 years of work experience (5203 teachers, 57.6%). More than half of the participants served as home-room teachers (5225 teachers, 57.9%) and most did not have prior experience in online teaching before the school hiatus (6387 teachers, 70.9%). In terms of subjects taught, most participants taught Chinese (2624 teachers, 29.05%), mathematics (2222 teachers, 24.60%) and English (1601 teachers, 17.73%). In order to analyze the representativeness of the sample, sample values for each demographic variable were compared to overall population statistics where available. Statistically significant but minor differences were found, with our sample being, on average, younger and more likely to report as female than the population average. It is also possible that younger teachers were more likely to respond to the online questionnaire or to be engaged in online teaching, as compared to teachers with more seniority. However, these differences, in terms of years or percentages, are small. Moreover, the large size of our sample (9030 participants) increases the likelihood of these differences being statistically significant without being of practical significance.

### 3.2. Descriptive Statistics and Pearson Correlations (RQ 1)

Table 2 displays the mean values for our target variables (observed scores) and Pearson correlations. For psychological distress, the mean was 0.36 (SD = 0.48). According to the cut-off points from the DASS-21 manual [28], the proportion of respondents with mental health problems were as follows: depression (20.4%), anxiety (26.4%), and stress (10.2%). 

The mean score for PSU was 2.76 (SD = 1.03), while the mean score for PSMU was 2.26 (SD = 0.79). More specifically, in terms of PSU, the proportion of respondents who scored over the cut-off value of 21, suggesting that an intervention was necessary [57], was 27.1%, while the proportion of respondents with problematic PSMU scores over 19 [58] was 13.1%, indicating that some levels of PIU were observed among respondents. 

Moreover, the mean value for fear of COVID-19 was 2.69 (SD = 0.75). The proportion of respondents reporting “agree” or “strongly agree” for fear of COVID-19 items, overall, was 33.83%, suggesting that roughly one third of respondents were fearful of COVID-19.

Overall, the mean score for PNT of online teaching was 3.54 (SD = 0.89). Among the three subscales of PNT, the scores from high to low were competence (mean = 4.30, SD = 1.38), autonomy (mean = 4.02, SD = 1.09), and relatedness (mean = 2.58, SD = 1.08) (not shown in Table 1). The proportion of respondents responding, on average, with “somewhat agree” (4 out of 7 on a Likert-type scale) or above accounts for 36.83% of respondents in terms of autonomy, 50.17% for competence, and 6.58% for relatedness. These results demonstrate that teachers’ psychological needs for competence were more strongly thwarted, as compared to the need for autonomy or relatedness. Repeated measures ANOVA determined that the difference among responses to these three subscales was significant: *F*(2,18058) = 8636.19, *p* < 0.001, with a large effect size *η_p_^2^* = 0.49 [65]. Bonferroni post hoc tests confirmed that PNT scores were significantly higher for competence thwarting as compared to the other two and significantly higher for autonomy thwarting as compared to relatedness.

Pearson correlations among measured variables indicated that psychological distress was positively associated with fear of COVID-19, PSU, PSMU, and PNT of online teaching (*r* values ranged from 0.25 to 0.41, all significant at *p* < 0.001). While significant, it should be noted that these coefficients are small and should be interpreted carefully as the relationships among variables based on correlation are not strong. 

### 3.3. Measurement Model (RQ 2)

Five latent variables and 25 indicators were included in the measurement model. The results indicated that the overall measurement model had acceptable fit, meeting the criterion: χ^2^ = 4909.45 (*p* < 0.001), CFI = 0.988, NNFI = 0.986, RMSEA = 0.044, and SRMR = 0.069. Moreover, regarding the discriminant and convergent validity, the results (see Table 2) showed that the square root of the AVE of each latent variable exceeded the correlations between other latent factors for each pair, that is, the discriminant validity was supported for each latent variable. The convergent validities for each latent variable were mostly supported. Specifically, all factor loadings for the indicators were above 0.50, AVE ranged from 0.44 to 0.90, and CR ranged from 0.70 to 0.96.

### 3.4. Full Structural Equation Model (RQ 2)

**Figure 2:**
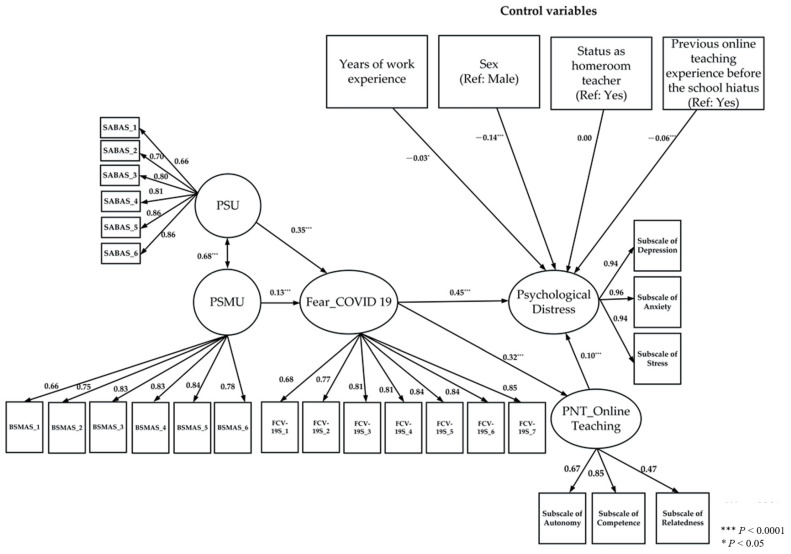
Structure equation model, including path coefficients.

Before testing the conceptual model, we first evaluated multicollinearity among the variables in the model. Variance inflation factors (VIF) were computed for each variable, which ranged from 1.11 to 1.61, suggesting that multicollinearity was not an issue in this study [66]. In the full structural equation model, the variables of years of work experience, sex, status as a home-room teacher, and previous online teaching experience before the school hiatus (when schools closed and online courses commenced) served as control variables (see Figure 2). The result demonstrated that the whole model had acceptable model fit (χ^2^ = 6440.42, CFI = 0.984, NNFI = 0.982, RMSEA = 0.043, and SRMR = 0.074). PSU and PSMU were positively associated with fear of COVID-19 (PSU: path coefficient = 0.35, *t* = 18.51, *p* < 0.001; PSMU: path coefficient = 0.13, *t* = 6.67, *p* < 0.001). The coefficient of fear of COVID-19 on PNT of online teaching was 0.32 (*t* = 19.62, *p* < 0.001) and on psychological distress was 0.45 (*t* = 8.99, *p* < 0.001). Moreover, PNT of online teaching had a direct effect on psychological distress (path coefficient = 0.10, *t* = 5.43, *p* < 0.001). Finally, PSU and PSMU had an indirect positive effect on psychological distress through the mediator of fear of COVID-19 and PNT of online teaching (PSU: path coefficient = 0.17, *t* = 8.99, *p* < 0.001; PSMU: path coefficient = 0.06, *t* = 5.65, *p* < 0.001). Lisrel syntax for the full structural equation model is provided in Supplementary Materials in Table S2.

### 3.5. Common Method Bias

Harman’s one-factor test was conducted to assess common method bias [64]. Five factors were extracted with eigenvalues greater than one. The total variance explained by the first factor was 32%, which was below the threshold of 50%. The other four factors contributed to a remaining 35% of the variance. This suggests that although there is likely to be some common method variance, the effect is not large. Thus, while common method bias cannot be totally ruled out as a contributing factor in this present study, it should not be a significant factor.

## 4. Discussion

Due to the COVID-19 outbreak, many schools around the world were temporarily closed, and governments had to implement online education in a short time frame. In consideration of the vulnerability of primary and middle school teachers who were restricted to their homes and required to conduct online teaching, the present study contributes to the literature as a unique empirical study modeling and investigating the mental health among primary and secondary school teachers under COVID-19. This study also utilizes a new instrument, adapted for a PNT scale for teachers to include the effects of online teaching on teachers’ PNT.

### 4.1. Principal Results

The results demonstrated that more than 70 percent of teachers had no experience with online teaching before the school closures, highlighting the fact that teachers did not receive adequate training in online teaching prior to the pandemic. Moreover, the results indicate that around 10–26% of participants suffered from mental health problems (from mild to severe), of which depression was the most serious. These results can be compared to previously reported findings among Chinese adults of an overall prevalence of anxiety of 13.67% among teachers, including primary, middle school, high school, and university teachers [9], and even earlier findings of only 4.98% in 2013 [67]. Potential reasons for this prevalence of reported mental health issues in the current study may be explained by the particularly vulnerable nature of primary and middle school teachers [9,10], particularly in the context of online teaching [6,14] and with increasing fear of COVID-19 being perpetuated by PSU and PSMU [27,29]. Moreover, teachers’ levels of psychological need thwarting (PNT) of online teaching differed according to need: *F*(2,18058) = 8636.19, *p* < 0.001, *η_p_^2^* = 0.49. Competence thwarting was much more prevalent than autonomy thwarting, with relatedness needs being the least thwarted, according to respondents. The role of competence thwarting in terms of online teaching and teacher psychology is supported by previous studies [6,7,8,10], which found that teachers perceived a lack of preparation and technological skills to handle online teaching. Teachers’ lack of time for interaction with colleagues during online teaching might have served as a buffer to reduce potential thwarting of relatedness needs due to less potentially negative interactions with colleagues or leaders (see the items for PNT of online teaching in Supplementary Materials Table S1). Likewise, the relatively moderate level of autonomy thwarting may be due to the fact that online teaching increased, out of necessity, teachers’ level of autonomy since teachers were required to develop course content independently.

### 4.2. Contextualization of Findings

It is interesting to note that level of the psychological distress among primary and middle school teachers in the present study was not as serious as originally expected, in that the scores were lower than those of a few studies of the general adult population during COVID-19 adopting the same instrument (i.e., DASS-21) [3,31,68]. We speculate that this difference may be attributed to two factors: (a) the time frame of the studies and (b) the countries in which the studies were conducted. In terms of the time frame, it should be noted that the study conducted by Wang et al. [68], which also involved respondents from China, took place between March and April 2020. As this period was at the peak of the epidemic in China, fear of COVID-19 was, arguably, much higher in comparison to responses collected by our study between the end of May to the end of June 2020. During the period evaluated by our study, the epidemic situation in China had eased considerably, which may have resulted in an overall lower level of fear of COVID-19, PSU, and PSMU. In terms of country, it should be noted that Boursier et al. [31] collected data in Italy during a lockdown in April 2020. The strong impact of the lockdown on the Italian population has been documented, with symptoms similar to those caused by trauma [69], whereas the response by Chinese citizens was less severe, with some research suggesting lockdown measures buffer the effects of social anxiety [12]. Likewise, Sigurvinsdottir et al. [3] included European and American respondents and data from April and May 2020.

In order to fully understand the mechanisms impacting teachers’ psychological distress, we developed and examined the conceptual model. Namely, the model considers PIU as a risk factor for psychological outcomes, such as fear, PNT of online teaching, and psychological distress [19,42,43]. This model focuses on psychological distress as the primary outcome, which results from fear of COVID-19 and PNT of online teaching, factors which are negatively impacted by PIU predictor variables, such as the psychological impact of negative online content through “(mis)information overload” [4]. 

### 4.3. Proposed Conceptual Model

Our results suggest that that excessive problematic Internet use behaviors should be considered as contributing risk factors in terms of teachers’ psychological distress, particularly in the online teaching context during COVID-19. In explaining the role of PIU among teacher populations, certain characteristics (i.e., excessive Internet use and workaholic tendencies) should be considered in the context of COVID-19 [70,71,72,73,74]. Specifically, teachers using the Internet for 5 or more hours per day are susceptible to the development of PIU [70]. In fact, during COVID-19, the time required by teachers for online teaching and preparation exceeded 5 h per day [71]. Moreover, teachers are a professional group at higher risk of workaholic tendencies, given the time required for teaching and preparatory activities [72,73], with workaholism considered a type of addiction [74,75]. Therefore, during the COVID-19 pandemic, teachers’ addictive workaholic tendencies, in addition to the excessive nature of required Internet use, are potential risk factors for PIU. Given the prevalence of PIU in this context, the impact of PSU and PSMU on teachers’ psychological well-being was more clearly observed.

Furthermore, given the requirements of online teaching, such as contacting parents and students online and preparing content for digital lessons, required teachers to frequently use smartphones and social media [10], which, given the negative nature of online content regarding COVID-19 [3,4,23,26], potentially harms teachers’ mental health by increasing fear of COVID-19 [27,29]. As such, from the perspective of SDT, the role of PNT of online teaching on teachers’ mental health is critical [41,47], with results indicating that PNT of online teaching is both influenced by fear of COVID-19 and predicts psychological distress.

In terms of the association between problematic Internet use behaviors and psychological distress, our results suggest the endogenous nature of PIU in contributing to psychological distress, mediated by fear of COVID-19. While this finding is consistent with previous studies [4,19,42,43,44,45,76], wherein excessive use of technology devices was harmful to individuals’ mental health, it contradicts some popular models which consider PSU and PSMU as exogenous variables in response to triggers such as stress [32,77]. However, further evaluation of potential reciprocal relationships should be conducted [78].

## 5. Conclusions

### 5.1. PIU as a Predictor

With data from more than 9000 primary and middle school teachers who engaged in online teaching during the COVID-19 outbreak, our large-scale survey contributes to the literature on the increasing “second wave” of psychological damage caused by COVID-19 [5] with validated and empirically supported findings. Our research suggests that, to a certain extent, primary and middle school teachers conducting online teaching may develop psychological problems, particularly when increased PSU and PSMU are contributing factors, although these effects are significant but relatively small. Our study echoes that of Aperribai et al. [10] who found that teachers restricted to their homes due to the epidemic had almost double the average scores on the Short General Health Questionnaire (GHQ 12), a screening tool for potential mental disorders, such as anxiety [79]. Findings from longitudinal research [78] further support the predictive role of PIU, particularly PSMU, in terms of psychological distress (anxiety and depression) for university students. As such, longitudinal research with teachers is an area for future evaluation.

### 5.2. The Impact of PNT of Online Teaching

Adopting the perspective of PNT [38,39] to interpret the phenomenon of decreased motivation for engaging in online teaching during the epidemic [7] is the main contribution of this study. Although the relationships are significant but small, these findings do contribute to our understanding of the role of PNT of online teaching in terms of teachers’ mental health outcomes. Stakeholders should carefully address the issue of teacher psychological need frustration, including autonomy, competence, and relatedness factors. Among these, lacking competence in conducting online teaching has been demonstrated as the main source of PNT, echoing findings highlighting teachers’ lack of sufficient skills as an obstacle to the implementation of online teaching [6,7,15]. Although competence frustration cannot be ameliorated in a short period of time (particularly during an epidemic), the autonomy and relatedness frustration may be alleviated by active interventions by school administrators. Principals are recommended to provide flexibility for online teaching, with stronger encouragement and active expression of care for teachers’ safety, mental health, and working status. By improving the online teaching environment to avoid thwarting teachers’ psychological needs, PNT may be significantly improved [35]. On the contrary, if school administrators maintain pre-epidemic management strategies without making corresponding adjustments (i.e., providing more autonomy and relatedness), PNT of online teaching could arise, subsequently harming teachers’ mental health.

### 5.3. PNT of Online Teaching as a Novel Research Construct

The potential of PNT of online teaching for future research and practice should be considered. The PNT of online teaching instrument was adapted by our team (including experts in health education, including professors and doctors), based on the original CPNTTS scale [35]. Items from the original scale were adapted in terms of wording in order to fit the context of online teaching. Great care was taken to ensure that the nature of the original items (in assessing the thwarting of autonomy, competence, and relatedness) was not undermined by revisions to the text. As such, in collaboration with primary and middle school teachers, face validity was established. Further analysis on the psychometric properties of the PNT of online teaching and the CPNTT scale demonstrated similar internal reliability and construct validity (including factorial, convergent, and discriminant validity). The factor loadings were similar for each item. While the instrument is still undergoing assessment of measurement invariance, we believe that further investigation and development of local (language or context-specific) scales measuring PNT caused by online teaching can provide benefits to researchers and practitioners evaluating the impact of online teaching on teachers’ psychological well-being.

### 5.4. Limitations and Contributions

Certain limitations of this study must be acknowledged. First, while all teachers included in our analysis were engaged in online teaching and, as such, required to use the Internet more frequently, it is possible that some teachers may have had PIU behaviors before online teaching began, a factor which should be evaluated in future studies in order to determine the role of PIU as a predictor in the context of online teaching. Secondly, regarding the influence of PSU and PSMU on psychological distress, although we have provided some explanation, causal relationships cannot be directly inferred from our data. As such, further qualitative data collection regarding teachers’ reasons for smartphone or social media use would add additional valuable interpretation. The findings from this study showed significant yet relatively small relationships among variables. As such, the selection of data collection instruments is critical for future research building upon these findings. The third limitation is related to sample representativeness. Since the timely collection of data was of key importance to this study, we conducted a rapid online survey using a non-random sampling frame. 

“Disrupted Classes, Undisrupted Learning” was the Chinese governments’ official policy in response to the COVID-19 outbreak [6]. While research has focused on mental health problems from the perspective of students [11,13,44], the vulnerability of primary and middle school teachers has received little attention. Our large-scale investigation endeavored to investigate psychological distress experienced by primary and middle school teachers in consideration of the acknowledged potential for PIU and our novel application of PNT of online teaching as key factors influencing psychological distress. Our findings suggest that PIU is strongly related to fear of COVID-19, which, in turn, impacts PNT of online teaching and psychological distress, with higher levels of PNT of online teaching associated with increased psychological distress. Based on these findings, we suggest that school administrators pay greater attention to teachers’ psychological needs through efforts to assist and enhance teacher autonomy and relatedness through interpersonal relationships while alleviating PNT arising from online teaching tasks. Our unique emphasis on the PNT of online teaching can also serve as a direction for further research and practical consideration, as the implementation of online teaching must bear in mind the potentially negative impacts of PIU.

## Figures and Tables

**Table 1:** Key characteristics of the participants of the study (*N* = 9030).

Demographic Variable	Category	Value	Overall Population Statistics; Test of Statistical Significance
Age in year; *M (SD)*		33.94 (8.81)	37.78; t = −24.85 (*p* < 0.001)
School type; *n* (%)			χ*^2^* = 1.66 (*p* = 0.20)
	Primary school	5838 (64.65%)	64%
	Middle school	3192 (35.35%)	36%
Sex; *n* (%)			χ*^2^* = 30.88 (*p* < 0.001)
	Male	2467 (27.3%)	30%
	Female	6563 (72.7%)	70%
Years of work experience; *n* (%)			χ*^2^* = 260.54 (*p* < 0.001)
	Under 5 years	2207 (24.4%)	21%
	6–10 years	1620 (17.9%)	15%
	11–15 years	1268 (14.0%)	17%
	16–20 years	1049 (11.7%)	16%
	Above 21 years	2886 (32.0%)	31%
Home-room teacher; *n* (%)			Not available
	Yes	5225 (57.9%)	
	No	3805 (42.1%)	
Prior online teaching experience; *n* (%)			Not available
	Yes	2643 (29.30%)	
	No	6387 (70.70%)	
Subject taught; *n* (%)			Not available
	Chinese	2624 (29.05%)	
	English	1601 (17.73%)	
	Mathematics	2222 (24.60%)	
	Science	716 (7.93%)	
	Social science	1008 (11.17%)	
	Other (music, art, physics, politics)	859 (9.52%)	

Sources: Age structure of primary and secondary school teachers [48]; population statistic from the Chinese Ministry of Education [49].

**Table 2:** Descriptive statistics and Pearson correlation matrix of the variables of interest.

	Mean (SD)	1	2	3	4	5
1. Psychological distress	0.36 (0.48)	1.00				
2. Fear of COVID-19	2.69 (0.75)	0.41 *	1.00			
3. PSU	2.76 (1.03)	0.34 *	0.35 *	1.00		
4. PSMU	2.26 (0.79)	0.31 *	0.27 *	0.57 *	1.00	
5. PNT	3.54 (0.89)	0.25 *	0.28 *	0.26 *	0.18 *	1.00

* Note: All *p*-values < 0.001.

## Supplementary Materials

The following are available online at www.mdpi.com/article/10.3390/healthcare9091199/s1, Figure S1: Scree plot for the Psychological Need Thwarting of Online Teaching Scale, illustrating the best fit for three factors; Table S1: Exploratory factor analysis (EFA) for the Psychological Need Thwarting of Online Teaching Scale (n = 4488), including a comparison with the original items from the Chinese Psychological Need Thwarting of Teachers Scale; Table S2: LISREL syntax for the proposed model.
