# Supplementary material for: The Effect of Primary and Middle School Teachers’ Problematic Internet Use and Fear of COVID-19 on Psychological Need Thwarting of Online Teaching and Psychological Distress
Source: Healthcare (Basel). 2021 Sep 11;9(9):1199. doi: 10.3390/healthcare9091199 (PMC8466317; doi:10.3390/healthcare9091199)
Supplement: Supplementary file 1 [file healthcare-09-01199-s001.zip › healthcare-1307049-SI.pdf]

Supplementary Materials

Table S1. Exploratory factor analysis (EFA) for the Psychological Need Thwarting of Online Teaching Scale (n=4488), including a comparison with the original items from the Chinese Psychological Need Thwarting of Teachers Scale.

Original Item: Chinese Psychological Need Thwarting of Teachers Scale	Revised Item: Psychological Need Thwarting of Online Teaching Scale	Factor Loadings after Promax Rotation		
		Relatedness	Competence	Autonomy
I feel disconnected from the people around me in my work environment.	9. I feel disconnected from other colleagues and leaders when teaching online during the pandemic.	**0.755**	0.079	0.020
I feel that people in my work environment are indifferent to me.	10. I do not feel that my colleagues and leaders care about me when teaching online during the pandemic.	**0.701**	0.182	-0.033
I feel that people around me are jealous of me when I am successful.	11. I feel that my colleagues and leaders are jealous of me when I achieve good results in online teaching during the pandemic.	**0.931**	-0.139	-0.011
I feel that people at school do not like me.	12. I feel that my colleagues and leaders do not like me when I conduct online teaching during the pandemic.	**0.948**	-0.152	-0.003
There are situations that make me feel incapable in my daily work environment.	5. There are some online teaching situations that make me feel incapable in my daily work environment during the pandemic.	-0.065	**0.795**	0.119
Sometimes I talk about the things that make me feel incompetent.	6. I sometimes talk about the things that make me feel powerless to do my online teaching job during the pandemic.	-0.016	**0.889**	-0.004
In my daily work, some situations make me feel powerless.	7. Online teaching during the pandemic sometimes makes me feel powerless.	0.027	**0.916**	-0.064
I feel that I am not capable of performing daily tasks due to the lack of opportunities for practice in my environment.	8. Due to the lack of training opportunities in my environment, I feel that I am capable of performing online teaching tasks.	0.456	**0.532**	0.071
I cannot decide for myself how I want to teach.	1. In online courses during the pandemic, I cannot decide for myself how I want to teach.	0.082	0.070	**0.519**
There is pressure to behave in a certain way in my	2. In online teaching work during the pandemic, I feel	0.022	0.100	**0.611**

daily work.	there is pressure that affects my behavior and requires me to comply in a certain way.			
I have to follow a certain prescribed teaching style.	3. I have to follow a prescribed online teaching style during the pandemic.	-0.066	-0.126	**0.822**
I feel pressure from external environment that limits me in choosing a particular teaching style.	4. During the pandemic, I feel pressure from the external environment that limited me in choosing a particular online teaching style.	0.008	0.078	**0.727**
	**Eigenvalues**	4.954	2.349	1.065
	**Percentage Variance**	0.246	0.201	0.158
	**Cumulative Percentage Variance**	0.246	0.447	0.605

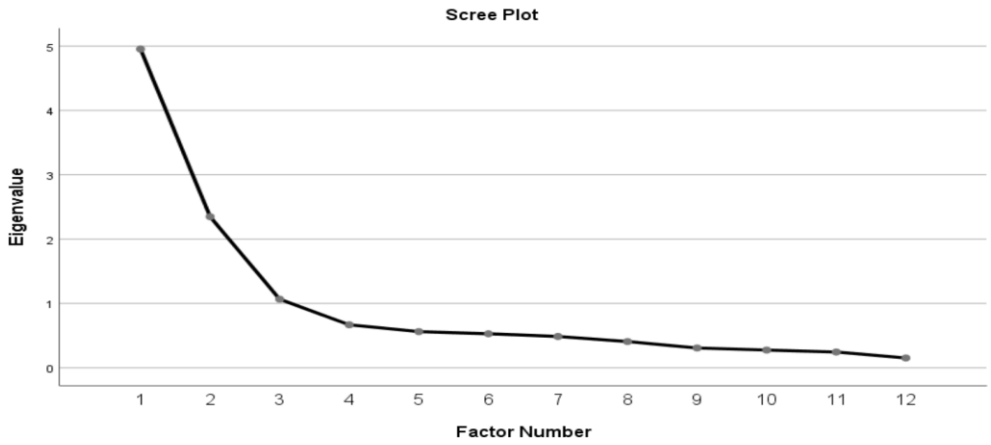

Figure S1. Scree plot for the Psychological Need Thwarting of Online Teaching Scale, illustrating the best fit for three factors.

Table S2. LISREL syntax for the proposed model.

```
DA NI=29 NO=9030 MA=PM
PM SY FI=1218_FOUR.PM
ACM SY FI=1218_FOUR.ACM
MO NY=13 NE=3 NK=6 NX=16 LX=FU, FI TD=SY, FI LY=FU, FI BE=FU, FI GA=FU, FI
FR LY 2 1 LY 3 1
FR LY 5 2 LY 6 2 LY 7 2 LY 8 2 LY 9 2 LY 10 2
FR LY 12 3 LY 13 3
FR LX 2 1 LX 3 1 LX 4 1 LX 5 1 LX 6 1
FR LX 8 2 LX 9 2 LX 10 2 LX 11 2 LX 12 2
VA 1 LY 1 1
VA 1 LY 4 2
VA 1 LY 11 3
VA 1 LX 1 1
VA 1 LX 7 2
VA 1 LX 13 3
VA 1 LX 14 4
VA 1 LX 15 5
VA 1 LX 16 6
FR TD 1 1 TD 2 2 TD 3 3 TD 4 4 TD 5 5 TD 6 6 TD 7 7 TD 8 8 TD 9 9 TD 10 10 TD 11 11 TD 12
12
VA 0 TD 13 13 TD 14 14 TD 15 15 TD 16 16
FR GA 2 1
FR GA 2 2
FR GA 1 3 GA 1 4 GA 1 5 GA 1 6
FR BE 1 2
FR BE 1 3
FR BE 3 2
LK
BSMAS SABAS WORKINGYEAR P_ONLINETEACHING_EXPERIENCE
HOMEROOMTEACHER GENDER
LE
DASS FEAR PNT
PD
OU ME=DWLS SS SC ND=3
```
